# Adult Plasticity in the Subcortical Auditory Pathway of the Maternal Mouse

**DOI:** 10.1371/journal.pone.0101630

**Published:** 2014-07-03

**Authors:** Jason A. Miranda, Kathryn N. Shepard, Shannon K. McClintock, Robert C. Liu

**Affiliations:** 1 Department of Biology, Emory University, Atlanta, Georgia, United States of America; 2 Center for Behavioral Neuroscience, Atlanta, Georgia, United States of America; 3 Graduate Program in Neuroscience, Emory University, Atlanta, Georgia, United States of America; 4 Institute for Quantitative Theory and Methods, Emory University, Atlanta, Georgia, United States of America; 5 Center for Translational Social Neuroscience, Atlanta, Georgia, United States of America; Rutgers University, United States of America

## Abstract

Subcortical auditory nuclei were traditionally viewed as non-plastic in adulthood so that acoustic information could be stably conveyed to higher auditory areas. Studies in a variety of species, including humans, now suggest that prolonged acoustic training can drive long-lasting brainstem plasticity. The neurobiological mechanisms for such changes are not well understood in natural behavioral contexts due to a relative dearth of *in vivo* animal models in which to study this. Here, we demonstrate in a mouse model that a natural life experience with increased demands on the auditory system – motherhood – is associated with improved temporal processing in the subcortical auditory pathway. We measured the auditory brainstem response to test whether mothers and pup-naïve virgin mice differed in temporal responses to both broadband and tone stimuli, including ultrasonic frequencies found in mouse pup vocalizations. Mothers had shorter latencies for early ABR peaks, indicating plasticity in the auditory nerve and the cochlear nucleus. Shorter interpeak latency between waves IV and V also suggest plasticity in the inferior colliculus. Hormone manipulations revealed that these cannot be explained solely by estrogen levels experienced during pregnancy and parturition in mothers. In contrast, we found that pup-care experience, independent of pregnancy and parturition, contributes to shortening auditory brainstem response latencies. These results suggest that acoustic experience in the maternal context imparts plasticity on early auditory processing that lasts beyond pup weaning. In addition to establishing an animal model for exploring adult auditory brainstem plasticity in a neuroethological context, our results have broader implications for models of perceptual, behavioral and neural changes that arise during maternity, where subcortical sensorineural plasticity has not previously been considered.

## Introduction

Temporal processing at early levels of adult human auditory centers can be altered by extensive perceptual or musical training [1]–[3] and language learning [4], [5]. The mechanistic basis for such improvements is not well understood. Animal studies using brainstem slice preparations have demonstrated that subcortical auditory nuclei are capable of both synaptic plasticity and intrinsic plasticity [6], [7]. Likewise, electrical stimulation of the auditory cortex has uncovered vast subcortical plasticity through corticofugal connections [8]–[11]. The extent to which such mechanisms may be utilized in naturally occurring behavior is unknown. Further, sound exposure is known to shape subcortical auditory processing during postnatal development, but much less is known about adult plasticity [12]–[14]. This reflects a general lack of model systems for exploring naturally occurring adult plasticity in early auditory stations [6]. Here, we use the mouse maternal communication model to explore whether experience-dependent plasticity occurs naturally in the subcortical auditory pathway and test whether estradiol contributes to plasticity mechanisms.

In the first two weeks of life, mouse pups produce vocalizations with peak spectral components between 2–80 kHz that elicit maternal behavior like nursing and pup retrieval [15]–[17]. These sounds gain behavioral significance for the mother during pup-care [18] and their neural representation is enhanced in the auditory cortex by improved temporal processing and inhibitory plasticity [19]–[23]. Whether subcortical auditory stations also express plasticity during motherhood is not known. If so, this would represent a novel, natural context for plasticity in the auditory periphery or brainstem, where neural changes mediated through physiological and/or experiential factors could be mechanistically investigated.

One leading hypothesis is that the maternal physiological state mediates improved auditory processing. A strong candidate mechanism is the hormone estrogen [24], for which blood plasma levels are elevated during pregnancy and return to low levels by about five days after parturition [25]. Estrogen facilitates pup call recognition by reducing the number of days of pup-care experience needed and may also modulate the perception of the acoustic features of calls [26], [27]. Estrogen alone does not improve recognition behavior but evidence from several vertebrate groups demonstrates that it modulates auditory processing, often in communication contexts [28]–[37]. This suggests that estrogen may act on auditory processing centers as part of the complex physiological transition to maternal behavior.

Pup-care experience, independent of pregnancy and post-partum hormones, might also result in improved temporal processing of sounds. Virgin mice, when paired with a mother, provide care for pups and eventually recognize pup isolation vocalizations as behaviorally relevant [26]. Such shifts could result from a change in the motivation to respond to calls, experience-dependent changes in the early sensory representation of calls that enhance neural processing in cocarers, similar to that observed in well-trained musicians [3], or a combination of increased behavioral relevance supporting experience-dependent plasticity. These considerations led us to explore whether and how experience-dependent, subcortical auditory plasticity can be induced in the mouse maternal communication system.

## Materials and Methods

### Ethics Statement

The Emory University Institutional Animal Care and Use Committee approved all procedures (permit number DAR-2000657-041114BN) under the standards set by the Animal Welfare Act, the Public Health Service Policy on Humane Care and Use of Laboratory Animals and the Association for Assessment and Accreditation of Laboratory Animal Care International.

### Animals

Experiments were carried out on adult female CBA/CaJ mice, predominantly between the months of March and June. All recordings from mothers and cocarers took place within one to 14 days after weaning pups (mean ± standard deviation (SD) of 5±5 days). Animals were housed under a reversed light cycle (14 hr light/10 hr dark), and had access to food and water *ad libitum*.

To test the role of maternal experience, the following animal groups were studied: mothers recently weaned of pups (n = 7, mean age ± SD 135±7 days), age matched pup-naïve virgins with no pup-care experience (n = 7, 134±10 days); and age matched cocaring virgin females with the duration of pup-care experience equal to that of the paired mother (n = 7,135±7 days). Furthermore, young virgins with no pup-care experience (n = 6, 80±4 days) were added to distinguish between different potential mechanisms for maternal effects.

To test the role of maternal levels of estrogen, we designed a treatment paradigm to approximate this hormone’s profile during and after pregnancy following similar protocols used in previous studies [26]. Virgin females with no pup-care experience were ovariectomized under 2–3% isoflurane and left for 13 days to allow hormones to clear. On day 14, one Silastic tube (0.078 in I.D.×0.125 in O.D.) implant was placed subcutaneously on the back of the animals. For eight females (mean age at implant ± SD, 84±5 days) the implant contained 50 µl of estradiol (β-estradiol, Sigma-Aldrich, St. Louis, MO) dissolved in sesame oil at a concentration of 3 mg/ml. The implant was sealed at the ends with silicone and soaked for 24 h in 0.9% saline before implantation. In a separate cohort of animals, we determined that this treatment results in a plasma estradiol concentration of (mean ± SD) 112±40 pg/ml (n = 3) as measured using radioimmunoassay by the Biomarkers Core Lab at Emory University. This concentration is comparable to levels reported in mice during the later stages of pregnancy [38], [39]. Eight control animals were ovariectomized and implanted with empty (“blank”) tubes sealed at both ends (mean age at implant ± SD, 83±5 days old). These animals were previously determined in a separate cohort to have plasma estradiol levels below the confidence limit of the radioimmunoassay at <22 pg/ml (n = 2).

### Auditory brainstem response

We recorded ABRs using Tucker Davis Technologies BioSigRP^©^ software running on a System 3 platform equipped with an RX5 Pentusa Base Station connected to subdermal electrodes via an RA4LI low impedance headstage (TDT, Alachua, FL, USA). We anesthetized mice with a single injection of ketamine and xylazine (100 and 10 mg/kg respectively) and placed them in a heated (25°C) sound attenuating booth (Industrial Acoustics Company, Bronx, NY) where the recordings took place. We limited the anesthetic to only a single injection to reduce the variation in anesthetic plane across animals that can occur after multiple injections. Sets of tone pips at different frequencies were presented in a random order to each animal to avoid an apparent frequency specific effect that may have been due to change in anesthetic plane. For example, a 16 kHz series was presented to one animal as the first stimulus and another animal as the third stimulus. This may have resulted in increased variation across animals for these stimuli. To reduce this variation in response to the click, we always characterized the click response at the end of the recording session. All animals were still fully anesthetized at this point.

A subdermal needle electrode was placed over the skull vertex as the active lead. The ground was placed ventral lateral to the left external pinna and the reference was placed ventral lateral to the right external pinna. The bioelectric signals were sampled at 25 kS/s, bandpass filtered between 100 Hz - 3000 Hz, amplified 200,000 times and averaged over 500 consecutive responses, following a previously established ABR screening protocol in mice [40]. To determine the ABR threshold, we reduced the stimulus intensity in 10 dB steps and then 5 dB steps until the lowest intensity at which the dominant ABR wave was visible. Since identifying peaks at threshold can be difficult, suspected focal peaks were required to be clear in multiple blocks of 500 averaged responses at the same stimulus intensity and patterns were compared to suprathreshold responses. This method is in line with common practice and previous studies of auditory thresholds in mice [40]–[42]. Thresholds were then finally determined offline with the observer blinded to an animal’s group identity. While we were always able to detect a threshold (i.e. sufficient speaker output), in some cases the blinded threshold was different than was estimated during recording. This resulted in missing latency data points, which are measured at 10 dB (tones) or 20 dB (clicks) above the blindly estimated threshold intensity, since responses to those higher intensities may not have been recorded. This can be noted from dot plots and the degrees of freedom in statistical analyses.

### Auditory stimuli

Calibrated stimuli were generated using TDT SigGenRP^©^ software and presented through BioSigRP^©^ software via a TDT RX6 digital signal processor at a sample rate of 195 kS/s. Sounds were attenuated by a TDT PA5 programmable attenuator and played free field from an Infinity EMIT tweeter placed 90° to the right side of the animal. Absolute sound pressure levels (SPL) of sound stimuli were measured prior to ABR recording experiments by placing a calibrated ¼” Bruel and Kjaer (B&K, Denmark) Type 4139 microphone with a Type 2669 preamplifier where an animal’s right ear would be located during playback. To measure the sound delivery system’s frequency-dependent transfer function, long duration pure tones were presented, recorded and amplified by a B&K Nexus conditioning amplifier Type 2690 connected to the TDT RX6 under the control of customized MATLAB (Mathworks, Natick, MA) calibration software provided by TDT.

During ABR recordings, positive-going broadband clicks of 0.5 ms duration were played back at a rate of 19 per second. Clicks presented at 0 dB of attenuation on the PA5 were measured using the calibration hardware described above at 99 dB SPL (re. 20 microPascals). A large proportion of the sounds produced by mouse pups, from immediately after birth until adulthood, are broadband or harmonic in structure [17]. These include sounds such as wriggling (primarily below 10 kHz), smacking, cracking, postpartum sounds while being cleaned by the mother after birth and sounds produced while being handled roughly by the mother. Pure tone pips of 3 ms duration with 1.5 ms cos^2^ rise/fall times were presented for 8, 16, 32, 64 and 80 kHz at a rate of 21 per second. These tone frequencies were chosen to span the previously reported spectral ranges of CBA/CaJ hearing [43] and the range of pup communication calls such as ultrasonic isolation sounds and lower frequency wriggling and rough handling sounds [16], [17]. Pure tone stimulus amplitudes were equalized using the above-measured speaker transfer function by adjusting the output voltage so that 0 dB of attenuation on the PA5 corresponded to an absolute measure of 74 dB SPL at each presented frequency. This was the physical limit of the system to produce high frequency tones.

### Data analysis

**Figure 1:**
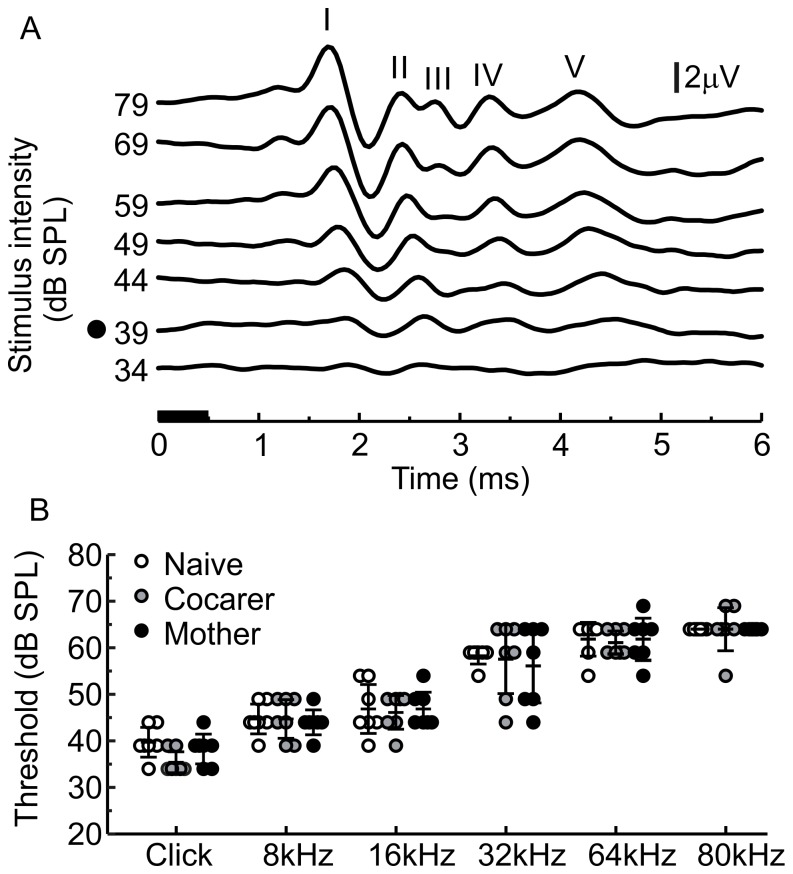
ABR thresholds for click and tone pip stimuli are not altered by maternal experience. A) Averaged click-evoked auditory brainstem responses (ABRs) (500 presentations per stimulus intensity). All traces are labelled with corresponding absolute stimulus intensity on the y-axis and a black dot denotes the threshold stimulus intensity. ABR peak numbers are labelled with roman numerals. The black horizontal bar on the x-axis denotes the duration of the stimulus. B) ABR thresholds for all stimuli. Dots represent ABR thresholds for individual animals with the middle horizontal bar representing the mean and error bars representing the 95% confidence interval.

Offline, after all ABR recordings, average ABRs were visualized and temporal features measured using BioSigRP^©^. Responses were coded to blind the observer to group identity. For click responses, we identified peaks I through V (Figure 1A) based on previously published conventions [44]. We compared groups on the absolute latencies for each peak as well as successive interpeak latencies. Testing the latter allowed us to localize plasticity in transmission between successive stages that could be independent of changes occurring at earlier auditory centers. For the tone pip-evoked responses, ABR thresholds over a smaller frequency range have been measured in mice previously [40], but response latencies have not. For this purpose, we defined peak I as the first peak that consistently appeared with low temporal variation upon repeated stimulation, a hallmark of ABR peaks [41]. Peak I was also chosen so that across different frequencies, it occurred within the range of the expected relative cochlear travelling wave delay [45]. The timing of the defined peaks in response to the click and tone stimuli were measured as the latency from the onset of the stimulus. When a peak consisted of two digital sample points with exactly the same amplitude value, the time of the earlier sample point was always taken as the peak latency.

To assess the effect of maternal experience on click and tone ABR latencies, a Multivariate Analysis of Variance (MANOVA) with Sidak correction for multiple post-hoc comparisons by ANOVA was done in SPSS 11.0. This was particularly important in enabling a more complete statistical analysis of the multiple dependent variables (different ABR peaks) as a function of the independent animal groups with varying levels of maternal behavior.

For the longitudinal study of the effects of estrogen manipulation on ABR latencies, we used linear mixed models to evaluate the overall effect of estradiol versus the blank implant. Two models were used for analysis of click-evoked data: one model used the five peak latency outcomes as the response variable, and the other model used the four interpeak latency outcomes as the response variable. In both models, time and outcome type (e.g., peak I vs. peak II) were treated as categorical variables. A separate model was used to analyze the tone-evoked data. This model evaluated the overall effect of estrogen versus blank on peak I latency while controlling for tone frequency and measurement time point (both as categorical variables). All models accommodated repeated measures made on the same mouse through a random intercept term that allows for correlation among measurements made on the same mouse. In all models, an interaction term between treatment group and time was included to allow for the treatment effect to be different at each time point. The interaction allows us to test our specific hypothesis that the effect of time on ABR latencies may differ in estradiol and blank treated animals.

Unless otherwise stated, statistical significance was evaluated at the α = 0.05 significance level.

## Results and Statistical Analyses

### ABR thresholds and maternal experience

We tested female mice for ABR thresholds in response to clicks and tones at 8, 16, 32, 64 and 80 kHz. ABR signal to noise ratio was higher for click responses when compared to tones as is expected when comparing a broadband stimulus to pure tone. Tone-evoked ABR quality is comparable to previously published examples [46]. Although 64 and 80 kHz tones are not commonly used for ABR threshold analyses, they fall within the range of ultrasonic vocalizations produced by mice [16] and therefore probe an ethologically relevant frequency range. Thresholds to tone stimuli in our study (Figure 1B) are higher compared to previous descriptions, most likely because we presented in an open field as opposed to closed field system. When comparing the three age-matched experimental groups, mothers, cocarers and pup-naïve virgin females did not statistically differ in mean ABR thresholds in a multivariate analysis across click and tone stimuli [MANOVA, *F* (12,26) = 0.465, p = 0.92, Wilk’s λ = 0.678], as is visually evident from Figure 1B.

### Click ABR latencies and maternal experience

We next examined whether having been a mother or caring for a sibling’s pups influences the temporal processing of sound. Mouse ABR peaks in response to the click stimulus are well described in the literature in terms of their latencies and the neural loci of their generation [44], [47]. Latencies for peaks I through V tended to be slower in our study than previous descriptions and, as with thresholds, was likely due to our open field presentation of the stimuli. We compared similarly aged mothers, cocarers and pup-naïve females on latencies to click-evoked peaks and the results from all comparisons and post-hoc analyses are presented in Tables 1 and 2.

**Table 1:** Click-evoked ABR peak latencies measured 20 dB above each animal’s threshold.

Peak	Mother	Cocarer	Naive	df	*F*	p
I	1.739±0.011^a^	1.763±0.025^a^	1.839±0.017^b^	2,18	7.839	0.004*
II	2.409±0.028^a^	2.474±0.031^a^	2.615±0.031^b^	2,18	7.831	0.004*
III	2.967±0.069	2.920±0.040	3.002±0.067	2,18	0.470	0.630
IV	3.561±0.153	3.337±0.051	3.578±0.085	2,18	1.635	0.223
V	4.219±0.195	4.142±0.049	4.471±0.116	2,18	1.655	0.220

Click ABR latencies (mean ± sem) at 20 dB above click threshold.

*Significant at the Sidak adjusted alpha level for multiple post-hoc ANOVA comparisons after a significant MANOVA: p = 0.010.

ab values sharing a common letter are not significantly different in a post-hoc analysis. All values are in milliseconds.

**Table 2:** Click-evoked ABR interpeak latencies measured 20 dB above each animal’s threshold.

Interpeak	Mother	Cocarer	Naive	df	*F*	p
I–II	0.670±0.022	0.711±0.019	0.776±0.039	2,18	3.674	0.046
II–III	0.558±0.053	0.447±0.017	0.388±0.038	2,18	4.984	0.019
III–IV	0.594±0.090	0.417±0.026	0.576±0.034	2,18	2.870	0.083
IV–V	0.658±0.070^a^	0.805±0.025^ab^	0.893±0.041^b^	2,18	5.916	0.011*

Click ABR interpeak latencies (mean ± sem) at 20 dB above click threshold.

*Significant at the Sidak adjusted alpha level for multiple post-hoc ANOVA comparisons after a significant MANOVA: p = 0.013.

ab values sharing a common letter are not significantly different in a post-hoc analysis. All values are in milliseconds.

**Figure 2:**
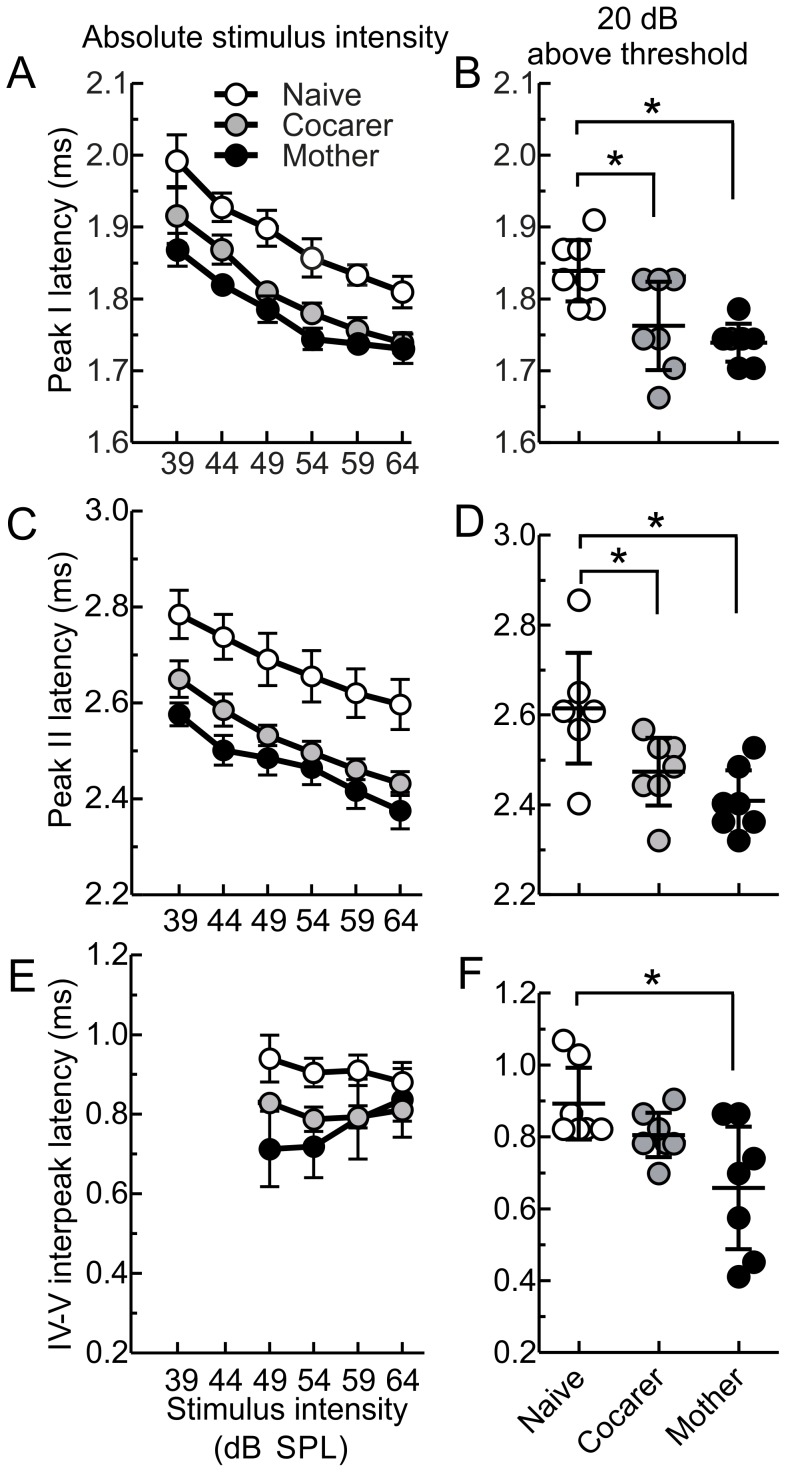
Click-evoked ABR latencies for specific peaks are significantly shorter for mothers than pup-naive females, with cocarers more similar to mothers. A) Latency to peak I at different absolute sound intensities (latency intensity function, LIF) and B) at 20 dB above ABR threshold. C) LIF for peak II and D) peak II latency at 20 dB above ABR threshold. E) LIF for interpeak latencies between peaks IV and V. No data points are shown for 39 and 44 dB SPL as this peak was not consistently present in all animals at lower stimulus intensities. F) interpeak latencies between peaks IV and V at 20 dB above auditory brainstem response threshold. LIFs in A), C) and E) show means with error bars denoting SEM. Dots in B), D) and F) show individual latencies for each animal with the middle bar representing the mean and error bars denoting 95% confidence intervals. Asterisks indicate statistical significance at p<0.010 in B) and p<0.013 in D) for the indicated post-hoc comparisons, using the Sidak adjusted alpha level.

Comparing the three groups, maternal experience significantly influenced mean ABR peak latencies with a significant multivariate effect for the five peaks [MANOVA, *F* (10, 28) = 3.416, p = 0.005; Wilk’s λ = 0.203]. Figure 2A shows latency intensity functions (LIF) for all three groups at peak I, demonstrating that mothers and cocarers were consistently earlier than pup-naïve females at each absolute sound intensity. Aligning relative to each animal’s click-evoked threshold, mothers and cocarers had significantly shorter latencies to peak I when compared to pup-naïve females for stimulus intensities 20 dB above threshold (Table 1, Figure 2B). This shorter latency was maintained in both mothers and cocarers at peak II (Table 1, Figure 2C, D). No other peak latencies showed significant differences among groups (Table 1) presumably due to the trend of increasing variability and width of each successive peak.

Maternal experience also influenced interpeak latencies with a significant multivariate effect for the four interpeak comparisons [MANOVA, *F* (8, 30) = 3.767, p = 0.004; Wilk’s λ = 0.249]. Figure 2E shows LIFs for all three groups at interpeak latency IV–V. Aligning relative to each animal’s click-evoked threshold, mothers had significantly shorter interpeak latencies between peaks IV and V when compared to pup-naïve females for stimulus intensities 20 dB above threshold (Table 2, Figure 2F). Cocarers showed intermediate latencies that were not significantly different from either mothers or pup-naïve females. No other interpeak latencies showed significant differences among groups (Table 2).

**Figure 3:**
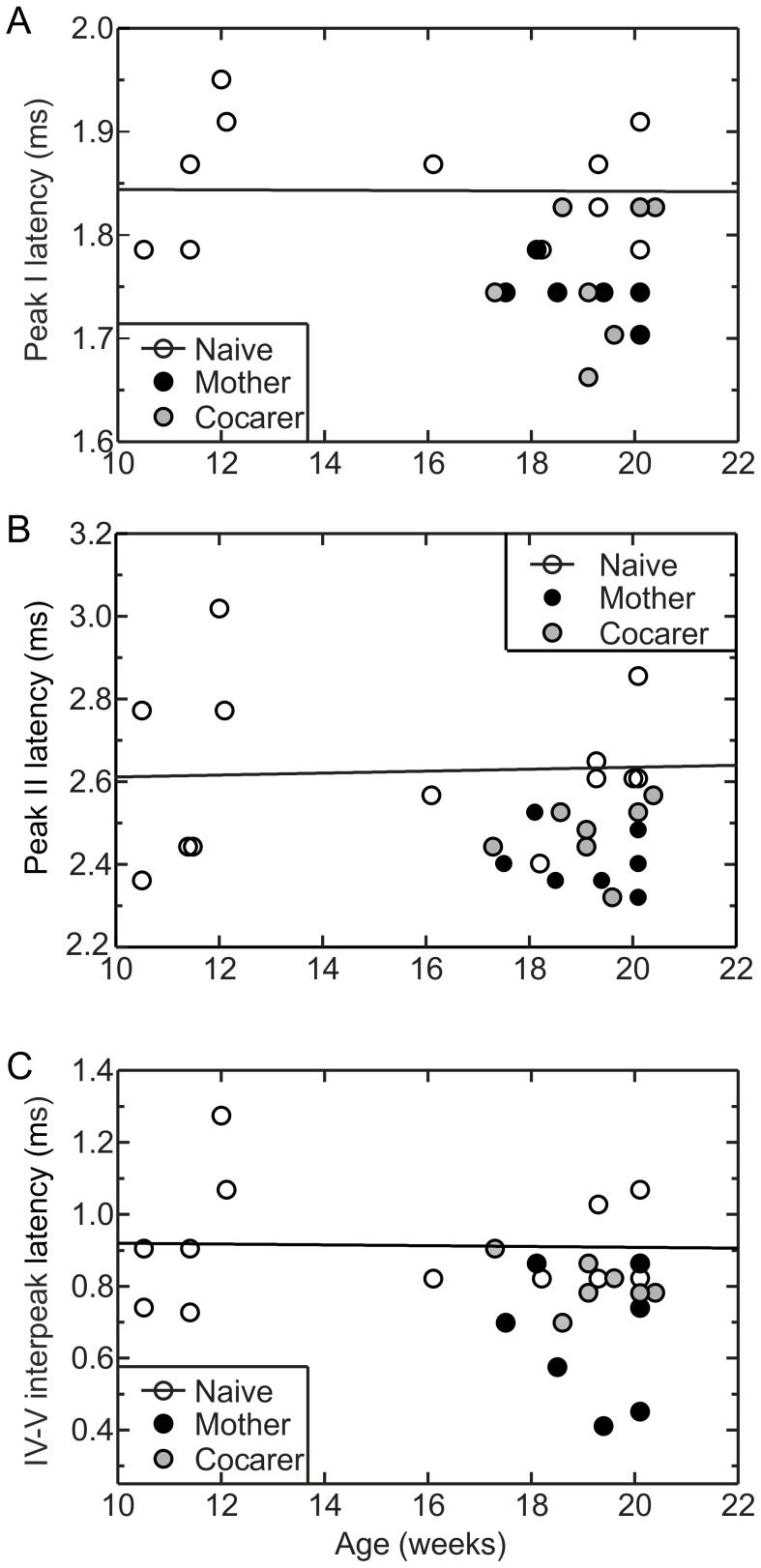
Shorter click-evoked ABR latencies in mothers represent a genuine shift towards faster processing. A) Peak I and B) peak II latencies, and C) IV–V interpeak latencies as a function of age, measured 20 dB above each animal’s threshold. For these peaks, which differed significantly between pup-naive females and mothers, pup-naive females did not show any age-related latency shift. Trend lines refer to pup-naive points only. r^2^ for all panels is <0.001. Data points for mothers and cocarers are all below these trend lines.

Shorter ABR latencies in mothers might be due to genuinely faster processing in mothers, or a protection from a general age-related slowing of processing [48] in virgins. To distinguish these, we measured ABR latencies in six younger pup-naïve females aged 10–12 weeks. This was the age at which virgin females were mated to become mothers in our study. We found no correlation between age and ABR Peak I latency among pup-naïve females [r^2^<0.001, p>0.05] (Figure 3A), with mean peak latencies remaining stable between 10 and 20 weeks of age. The same was true for peak II latency [r^2^<0.01, p>0.05] (Figure 3B) and for interpeak latencies between peaks IV and V [r^2^<0.001, p>0.05] (Figure 3C). This suggests that the shorter ABR latencies observed after motherhood likely represent a shift towards faster processing.

### Tone ABR latencies and maternal experience

**Figure 4:**
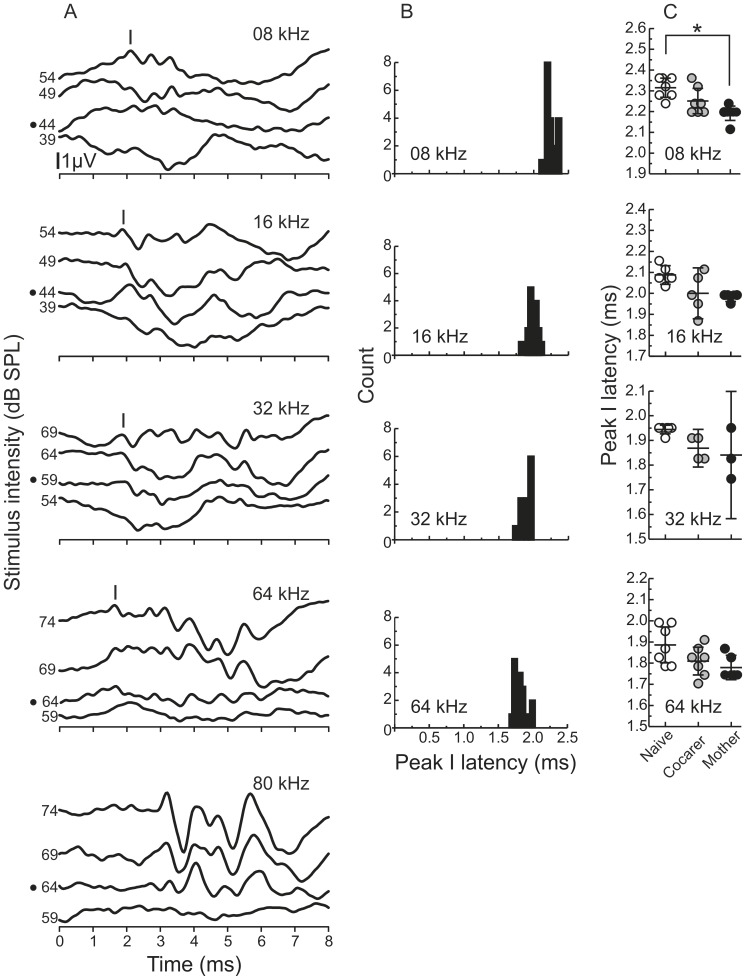
Maternal experience significantly influences tone pip-evoked ABR peak latencies. A) Representative averaged traces from individual animals in response to tone pips at different frequencies (500 presentations per stimulus intensity). The black dot to the left of the y-axis denotes the threshold stimulus intensity. Peak I is labelled in each auditory brainstem response with the exception of 80 kHz as we were unable to identify a Peak I latency at this frequency. B) Distributions of latencies for all animals combined. Latencies measured 10 dB above each animal’s threshold. Bin width = 0.02 ms C) Effects of pup-care experience on peak I latencies. Dots show individual latencies for each animal with the middle horizontal bar representing the mean, and error bars denoting 95% confidence intervals. Asterisk indicates significance at the p<0.01 for the indicated post-hoc comparison.

As an additional test of temporal differences in early auditory processing across animal groups, we also examined tone pip-evoked ABR latencies. To our knowledge, ABR peak latencies in response to tone pip stimuli have not been previously described in depth, and the precise mapping of peaks to neural generators is not well established. Hence, here we focused only on identifying the *first* dominant peak in the ABR response to 8, 16, 32 and 64 kHz tone pips (Figure 4A), which presumably reflects similar neural processes as those underlying the click-evoked peak I. For each frequency, this peak was chosen for its consistent appearance in all animals and for its expected relative latency based on the cochlear traveling wave delay [45]. The ranges for peak I latencies at 10 dB above threshold, when considering all females in the maternal study together, were 2.115–2.362 ms for 8 kHz, 1.869–2.156 ms for 16 kHz, 1.745–1.951 ms for 32 kHz and 1.740–1.992 ms for 64 kHz. The distributions of these peak latencies are shown in Figure 4B, which clearly illustrates the expected decrease in latency as tone pip frequency increases. We were unable to identify a consistent peak I within the expected latency range for the 80 kHz stimulus (Figure 4A) although longer latency peaks were prominent and consistent allowing for threshold determination at this frequency.

Latency differences among mothers, cocarers and pup-naïve females were not limited to clicks. We compared these groups on latencies to the first tone pip-evoked peak for the four frequencies characterized above, measured 10 dB above each animal’s threshold. Maternal experience significantly influenced tone pip-evoked ABR peak I latencies with a significant multivariate effect for the four frequencies [MANOVA, *F* (10, 28) = 3.416, p = 0.005; Wilk’s λ = 0.203]. Results from the post-hoc analyses are presented in Table 3. Mothers consistently showed faster latencies compared to naïves, with cocarers showing intermediate latencies at all frequencies. Post-hoc analyses at each tone frequency revealed statistical significance for 8 kHz.

**Table 3:** Tone-evoked peak I ABR latencies measured 10 dB above each animal’s threshold.

Tone frequency	Mother	Cocarer	Naïve	df	*F*	p
8 kHz	2.192±0.014^a^	2.251±0.025^ab^	2.315±0.019^b^	2,18	9.727	0.001*
16 kHz	1.984±0.001	2.000±0.044	2.088±0.017	2,13	4.677	0.030
32 kHz	1.841±0.060	1.869±0.024	1.944±0.007	2,10	4.360	0.043
64 kHz	1.779±0.022	1.810±0.027	1.886±0.035	2,17	3.575	0.051
80 kHz	NI	NI	NI			

Tone ABR latencies (mean ± sem) at 10 dB above tone threshold.

*Significant at the Sidak adjusted alpha level for multiple post-hoc ANOVA comparisons after a significant MANOVA: p = 0.013.

ab values sharing a common letter are not significantly different in a post-hoc analysis. All values are in milliseconds. NI, peak I not identifiable at this frequency.

### Simulating a maternal time course of estradiol alone cannot explain maternal ABR plasticity

**Figure 5:**
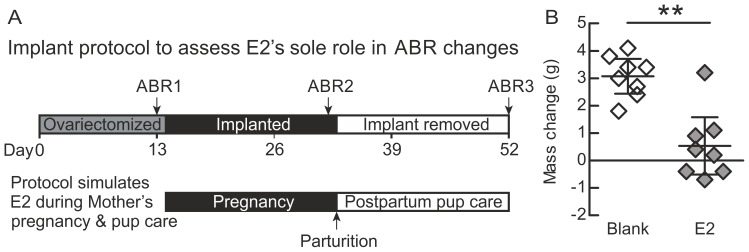
Time course and efficacy of hormone manipulation. A) Timeline of estradiol treatment and auditory brainstem response recordings in relation to the mouse pregnancy and maternal care timeline. B) Change in mass from the beginning of estradiol treatment to the end verifies the expected reduced weight-gain from elevated estradiol levels. Middle bars represent the means; error bars represent 95% confidence intervals.

Given the generalized effects of motherhood on the ABR, we next considered whether some aspect of the intrinsic physiological state induced by maternity may have been responsible. We focused on the potential role of estrogen, which is a key hormone whose plasma level reaches a sustained, high level during pregnancy [25]. Estrogen is thought to help prime neural circuits in advance of parturition so that mothers will be more receptive to cues from their young [49]–[51]. To determine whether the temporal profile of maternal estrogen could account for the improvements seen in mothers, we determined ABR peak latencies at three time points (Figure 5A): (1) after an initial ovariectomy depleted gonadally produced estrogen (ABR 1); (2) after an estradiol implant had been in place for the duration of a typical pregnancy (ABR 2); and (3) after removing the implant, simulating the drop in estrogen at birth (ABR3). The last time point was taken after the typical duration of pup care following birth, to match the time point at which our natural mothers were studied.

To monitor the effectiveness of implants in experimental animals without the need to collect large blood samples, we documented the previously reported estradiol-induced anorexia in rodents [52], [53] by measuring body mass before implant and at the end of estrogen treatment. Blank implanted animals, who received empty implants, showed significantly greater weight gain during the treatment period when compared to those implanted with estradiol [t(14) = 4.907, p<0.001] (Figure 5B).

**Figure 6:**
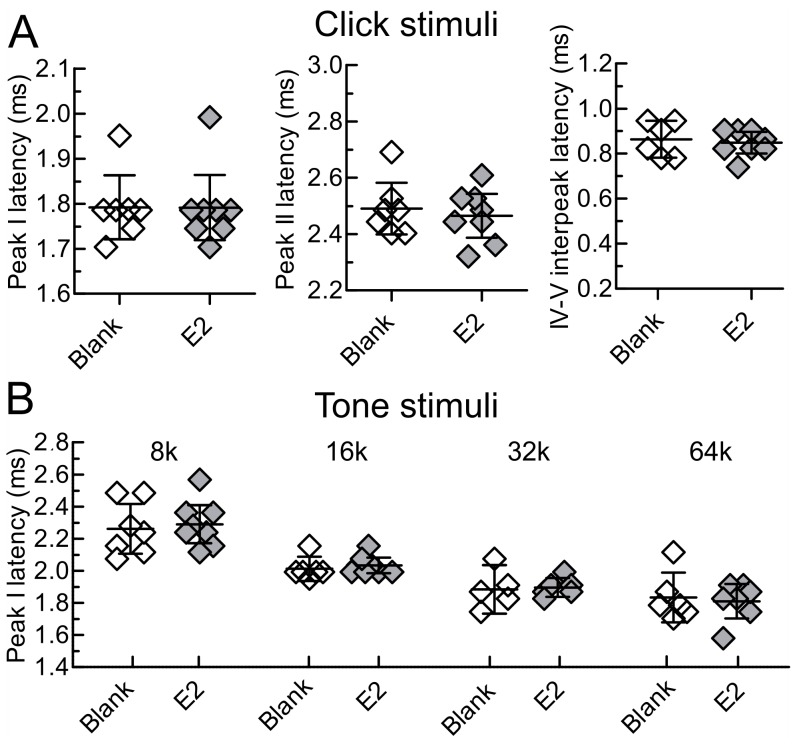
Maternal profile of estradiol does not significantly affect ABR peak latencies. A) Auditory brainstem response (ABR) latencies in response to click stimuli at 20 dB above threshold, at time point ABR3 (∼3 weeks after implant removal). The specific peaks shown are those that differ between mothers and pup-naïve females in the maternal study. B) ABR latencies in response to tone pip stimuli at 10 dB above threshold, at time point ABR3 (∼3 weeks after implant removal). Diamonds represent individual animals with the middle bar representing the mean. Error bars represent 95% confidence intervals.

We generated two linear mixed models to test our hypothesis that estradiol implantation would have a consistent affect on ABR peak and interpeak latencies. The peak latency model was based on 234 measurements from 5 peak latencies made on 8 mice in each of the estradiol and blank groups, while the interpeak latency model was based on 186 measurements from 4 interpeak latencies made on 8 mice in each group. Due to time constraints during the ABR recording, not all mice were measured at each time point for each outcome resulting in missing data points shown in Figure 6. Missing measurements were assumed to be missing at random.

The results of the mixed model are described below, and complete descriptive statistics for this study are presented in Tables 4, 5 and 6. For peak latency outcomes, there was no significant time point by treatment interaction [F(2,210) = 0.53, p = 0.59]. Moreover, no significant differences were found in mean latency between the blank and estradiol groups at time points ABR1 [t = 0.77, df = 210, p = 0.44], ABR2 [t = 1.48, df = 210, p = 0.14], or ABR3 [t = 0.85, df = 210, p = 0.39]. For the interpeak latency outcomes, again there was no significant time point by treatment interaction [F(2,163) = 0.24, p = 0.79]. Further, no significant differences were found in mean latency between the blank and estradiol groups at time points ABBR1 [t = 0.99, df = 163, p = 0.32], ABR2 [t = 1.66, df = 163, p = 0.10], or ABR3 [t = 0.82, df = 163, p = 0.42]. In particular, this null effect was illustrated in Figure 6A for peaks I and II, and interpeak IV–V at the “simulated” post-weaning time point ABR3, in which latency differences had been observed between maternal and nonmaternal animals (Figure 2).

**Table 4:** Estradiol effects on click-evoked ABR latencies measured 20 dB above each animal’s threshold.

Peak	Group	ABR1	ABR2	ABR3
I	E2	1.802±0.025	1.781±0.027	1.791±0.031
	Blank	1.817±0.035	1.797±0.028	1.792±0.029
II	E2	2.537±0.047	2.475±0.049	2.465±0.033
	Blank	2.506±0.047	2.496±0.050	2.491±0.038
III	E2	3.144±0.062	3.118±0.088	2.964±0.033
	Blank	3.041±0.053	3.041±0.059	2.920±0.081
IV	E2	3.689±0.158	3.756±0.159	3.493±0.121
	Blank	3.647±0.125	3.457±0.078	3.240±0.059
V	E2	4.470±0.141	4.450±0.173	4.341±0.113
	Blank	4.295±0.141	4.146±0.106	4.125±0.060

Click ABR latencies (mean ± sem) at 20 dB above click threshold. All values are in milliseconds.

**Table 5:** Estradiol effects on click-evoked ABR interpeak latencies measured 20 dB above each animal’s threshold.

Interpeak	Group	ABR1	ABR2	ABR3
I–II	E2	0.735±0.031	0.694±0.027	0.674±0.032
	Blank	0.689±0.036	0.699±0.025	0.699±0.016
II–III	E2	0.607±0.044	0.643±0.064	0.499±0.042
	Blank	0.535±0.040	0.545±0.046	0.429±0.050
III–IV	E2	0.545±0.102	0.638±0.075	0.530±0.101
	Blank	0.607±0.091	0.417±0.072	0.391±0.033
IV–V	E2	0.781±0.049	0.694±0.085	0.848±0.020
	Blank	0.648±0.063	0.689±0.089	0.864±0.032

Click ABR interpeak latencies (mean ± sem) at 20 dB above click threshold. All values are in milliseconds.

**Table 6:** Estradiol effects on tone-evoked peak I ABR latencies measured 10 dB above each animal’s threshold.

Tone frequency	Group	ABR1	ABR2	ABR3
8 kHz	E2	2.329±0.020	2.198±0.054	2.290±0.050
	Blank	2.239±0.049	2.229±0.023	2.262±0.063
16 kHz	E2	2.013±0.021	1.999±0.039	2.033±0.021
	Blank	2.000±0.044	2.018±0.037	2.013±0.029
32 kHz	E2	1.869±0.063	1.804±0.038	1.896±0.023
	Blank	2.013±0.144	1.841±0.017	1.885±0.055
64 kHz	E2	1.869±0.114	1.868±0.065	1.810±0.044
	Blank	1.904±0.070	1.921±0.078	1.834±0.060

Tone ABR latencies (mean ± sem) at 10 dB above tone threshold. All values are in milliseconds.

We took a similar approach to investigate the impact of estradiol on tone pip-evoked ABR latencies, generating a linear mixed model based on 150 measurements made in 8 mice from each of the estradiol and blank groups. As in the maternal study, we focused only on the first dominant peak of the ABR waveform. There was no significant time by treatment interaction [F(2,127) = 0.12, p = 0.88]. Hence, no significant differences were found in mean peak I latency between the blank and estradiol groups at time points ABR1 [t = 0.29, df = 127, p = 0.77], ABR2 [t = 0.53, df = 127, p = 0.60], or ABR3 [t = 0.08, df = 127, p = 0.94] (Figure 6B).

Taken together, the results of our comparison between estradiol and blank animals indicate that a maternal profile of estradiol alone cannot account for any of the observed differences in ABR latencies between mothers and pup-naïve females. Interestingly though, Table 4 shows an overall decrease in ABR peak latencies across time points in both experimental groups, most notably at peak IV. Indeed, across peaks, click-evoked peak latencies decreased significantly in both the estradiol [F(2,210) = 4, p = 0.02] and blank [F(2,210) = 3.5, p = 0.03] groups. However, the fact that this effect was observed both in animals that did and did not experience the estrogen fluctuation characteristic of maternity precludes the possibility that this effect could explain the shortened latencies present in mothers.

## Discussion

There is increasing evidence that the adult auditory periphery and brainstem are far more dynamic in their representation of sounds than previously appreciated [6]. On short time scales, efferent pathways are thought to enable transient changes in subcortical activity that mediate attentional effects on hearing [54]–[58]. On long time scales, peripheral damage increases spontaneous activity in the cochlear nucleus [59]–[62] and alters inhibitory neurotransmission [63], phenomena that may contribute to perceptual hearing disorders like tinnitus [64], [65]. In healthy human adults, a growing literature also indicates that long-term alterations in brainstem temporal processing arise as sounds become behaviorally relevant through extensive explicit or implicit training [1]–[5]. Our results in mice provide a new example of long-term subcortical plasticity in temporal processing in the context of a natural life experience in adulthood - motherhood. We found that the latencies of the earliest peaks in sound-evoked ABRs are generally shortened in maternal compared to virgin mice, without changes in ABR thresholds. Mechanistically, experience with pups, rather than simply the levels of a key maternal hormone, estrogen, must contribute to this, since cocaring virgins, but not estrogen-implanted virgins, show ABR latencies more systematically similar to mothers.

The neuroanatomical sources of the observed plasticity can be deduced from comparisons with earlier characterizations of click ABRs in mice and other species [44], [47], [66]. Changes in peak I latency indicate that temporal processing plasticity likely occurs at the level of the VIII-th nerve or the ear. Peak II emanates from the cochlear nucleus. The improved temporal response was maintained through to the cochlear nucleus as shown by the shorter latencies observed in mothers and cocarers at peak II. This could arise by either maintenance of the fidelity along this part of the auditory pathway or a second level of plasticity in the transmission to or responsiveness of the cochlear nucleus, in addition to changes occurring at the neural generators of peak I. The lack of a significant difference for the I–II interpeak latency suggests that the former is more likely. Lastly, shorter interpeak latencies in mothers between peaks IV and V suggest yet another level of plasticity likely to be located in the inferior colliculus. Given that humans also show experience-dependent improvement in temporal processing at the level of the inferior colliculus [2], this result is of particular interest as a future opportunity to begin studying the mechanisms of such plasticity.

Our results suggest that something about the maternal experience leads to changes in subcortical auditory processing in mice. One obvious factor is the hormonal change associated with pregnancy and parturition. Given that estrogen levels rise dramatically during pregnancy and parturition, and that estrogens have a documented role in modulating auditory processing [31], [32], [34], [35], [37], we initially hypothesized that elevated estrogens alone could affect ABR peak latencies. This would also be consistent with studies of older, postmenopausal women showing that long term estrogen replacement therapy shortens ABR peak latencies [28], [67], [68]. However, we found no evidence that either prolonged elevated estradiol or estradiol-withdrawal changes the ABR in our ovariectomized young adult mice. Our results differ from a previous study in rats [30] in which conjugated estrogens (Premarin) caused a reduction in ABR latencies for peak I and interpeak latency I–II. This may have been due to the difference in the form of estrogen replacement (Premarin vs. 17β-estradiol), the study organism, or our considerably longer treatment timeline, which was chosen to more closely mimic the estrogen changes occurring during the course of pregnancy and parturition. Furthermore, since sample sizes in both the rat and our studies were relatively small, the discrepancy motivates future follow-up studies with larger cohort sizes. We note though that for the purposes of this paper, our sample size should have been sufficient to reveal whether estradiol alone can account for the effect of motherhood on ABR latency, given that the cohort of maternal animals in which we observed the original effect was of comparable size and variance.

While our hormone manipulations were insufficient to mimic the effect of motherhood on ABR peak latency, we did note a significant decrease in click-evoked peak latencies across the three measurement time points in both estradiol- and blank-implanted mice. This may be an effect of age, though it is unlikely, as we demonstrated in a separate cohort of animals that peak latencies are relatively stable over the age range tested (Figure 3), in agreement with a prior study [69]. Alternatively, the overall shortening effect may be a lasting result of ovariectomy, since both estradiol- and blank-implanted animals were ovariectomized prior to ABR measurement at time point ABR1. This would also be unusual, as estrogen withdrawal by ovariectomy is not associated with decreases in ABR latency [30]. To conclude whether ovariectomy was responsible for this phenomenon would have required a gonadally intact longitudinal group as a control, which we did not include for statistical efficiency. Regardless, the lack of interaction between animal group and time in our study suggests that replacing only estradiol after ovariectomy is not sufficient to result in shorter ABR latencies. Whether estradiol is necessary in combination with maternal experience for shorter ABR latencies remains in question. Behavioral data suggest that it is not necessary for pup retrieval in females with pup-care experience [26].

Maternal levels of other hormones, such as progesterone or prolactin might instead contribute to shortening latencies in our maternal cohort, but much less is known about their role in audition. Progesterone tends to have more deleterious effects on peripheral hearing [70], possibly by blunting a protective effect attributed to estrogens [24]. However, progesterone does also promote myelination [71], and temporal changes in wave I might reflect increased myelination in the auditory nerve. Indeed, the ABR peak I latency is shorter in mice with greater peripheral myelin integrity [72]. Prolactin may also play a part in such a mechanism, since it regulates new oligodendrocyte production and increases the number of myelinated axons during pregnancy [73].

An alternative hypothesis to explain shorter ABR latencies in mothers is that exposure to the sounds from and/or care of vocalizing pups may drive experience-dependent changes in the auditory system. We found support for this hypothesis by assessing ABRs in cocaring virgins. The click-evoked peak I and II latencies were significantly shorter in cocarers compared to pup-naïve females, and comparable to that in mothers. Further along the auditory pathway, the IV-V interpeak latencies in cocarers were intermediate between, though not significantly different from, pup-naïve virgins and mothers, whose latencies were themselves significantly different. The same was true for peak I in response to 8 kHz tones, and a similar trend was observed for peak I in response to the other octave-spaced tones up to 64 kHz. The strongest effects were seen in response to broadband and lower frequency stimuli which are similar to well characterized sounds produced by pups such as wriggling, smacking, cracking and rough handling sounds [17]. Together these results suggest that pup care experience, independent of pregnancy and parturition, provides one impetus resulting in earlier responses in the subcortical auditory system. It may be that the extended experience with the range of vocalizations from pups [16], [74] promotes generalized plasticity in early auditory processing across the frequency spectrum so that it is most evident when tested with broadband stimuli like clicks. We therefore hypothesize that the shift in temporal processing in maternal mice is a similar phenomenon to the plasticity in the frequency following response of auditory brainstem activity after acoustic training in human adults [2]–[5]. If so, experience with the vocalizations of conspecific (or even one’s own) pups might produce higher fidelity ABR responses than vocalizations of another mouse strain or litter, which can differ in their acoustic characteristics [16], [75]. An important point though is that our study does not differentiate between passive acoustic exposure-induced plasticity, and plasticity arising from active social interactions with pups; future studies will need to address this.

Despite a demonstration for the role of experience, it alone does not completely explain the differences between mothers and pup-naïve females. This leaves open possibilities to systematically explore the combined roles of hormonal changes with vocalization and pup experience. In particular, a future study could longitudinally follow individual females through motherhood or cocaring itself. This would allow us to more tightly track how temporal processing changes with endogenous hormonal fluctuations during pup experience. Such a within-subjects design would also rule out the possibility that our current results are due to an unknown, systematic intrinsic difference between successful mothers that reach weaning (∼74% of mated females in our CBA/CaJ colony at Emory) and those either not picked for mating (virgins), or those that did not care well for pups (e.g. cannibalize their litters). A prospective design like this with larger sample sizes would be informative now that an effect at the post-weaning time point has been observed. It also illustrates a practical advantage of rodent models over human studies to reveal mechanisms of auditory plasticity during maternity or through the lifespan.

What might a reduction in ABR peak latencies mean to the function of the auditory system and the behavior of the animal? The magnitude of the latency difference is small (∼100 µs between mothers and naïve females), though significant and likely physiologically appreciable. For perspective, this is on the same order as the mouse’s interaural time difference for a lateralized sound [76], making such a size shift potentially relevant for processing by higher-order auditory stations. The accurate representation of time is a critical aspect of general auditory processing for sound localization, onset/offset detection, sound duration and amplitude modulation [77], [78]. For mice, temporal characteristics of different classes of communication sounds are important for recognition and categorical perception, particularly in the maternal context [79]–[81]. Shifts in the encoding of temporal characteristics of sounds could in principle shift the boundaries that define such categories for recognition and perception.

A physiological example of shifts in temporal responsiveness to communication signals is seen in the auditory cortex of mother mice, and subcortical changes might contribute to such persistent cortical modification. The ability of auditory cortical neurons to respond to individual pup isolation calls within a bout is improved in mothers when compared to pup-naïve females [20]. Mothers also show faster timing of cortical spiking that leads to increased information transmission in the neural responses of anesthetized mice [21]. Given the well established capacity for experience dependent plasticity within the auditory cortex [82], [83], it is likely that changes occurring during maternal care have a basis within the cortex. Behaviorally relevant cortical plasticity requires concurrent input from subcortical auditory regions and one of several neurotransmitter systems that are activated during maternal behavior [25], [84], [85]. Therefore, shifts in auditory processing in subcortical nuclei that coincide with pup-care experience, as suggested by our study at a time point several weeks after peak calling by pups [17], are likely to shape higher order auditory processing and thus behavior.

Subcortical auditory nuclei do exhibit plasticity mechanisms that could potentially alter spike timing in response to repeated exposure to auditory stimuli. For example, long-term potentiation can drive reduced spike latencies after recurring presynaptic activation, as has been shown in the hippocampus after repeated traversal of spatial receptive fields [86]. Both the cochlear nucleus and inferior colliculus of the mouse are capable of long term potentiation [6], [7], although *in vitro* studies investigating this mechanism are normally conducted in developing animals rather than adults.

The impetus for subcortical changes could actually arise from cortical activity via corticofugal modulation of early auditory processing during hearing experience, which then translates over repeated experience into a persistent improvement. For example, electrical stimulation of mouse primary auditory cortex facilitates shortened response latencies in cochlear nucleus neurons [9], [10], where we see latency shifts in mothers. A large body of evidence has demonstrated that corticofugal modulation of the inferior colliculus, where we again see latency shifts in mothers, shapes neural sensitivity to sound frequency and intensity but much less is known about the temporal domain [8], [11], [87], [88]. One exception is in several species of bats, where electrical stimulation of the auditory cortex does improve temporal processing but little is known about how much this occurs outside this specialized group of mammals and its role in modulating naturally occurring behavior [89]. This issue, which is pertinent to adult auditory plasticity in humans [6], is well suited for dissection in an animal model of natural adult plasticity.

Finally, our study implicates early auditory processing as a new addition to previously well established models of the neural circuitry regulating maternal responsiveness. Traditionally, these models focus on motivational gating of behavioral responses to sensory stimulation by infants, controlled by the medial preoptic area of the hypothalamus [50]. More recently, changes in the parental perception of infant cries have also been found, but attributed to differences mainly in the amygdala, anterior cingulate cortex, and other forebrain regions [90], [91]. Our results suggest that changes in perception may ultimately originate from plasticity in how infant vocalizations are encoded by subcortical auditory stations as a result of maternal experience [92]. Thus, to more completely understand the neural basis for how communication signals engage natural behaviors, such sensory plasticity should be incorporated into future models of maternal responsiveness.
